# Comparison of Silks from *Pseudoips prasinana* and *Bombyx mori* Shows Molecular Convergence in Fibroin Heavy Chains but Large Differences in Other Silk Components

**DOI:** 10.3390/ijms22158246

**Published:** 2021-07-31

**Authors:** Michal Rindos, Lucie Kucerova, Lenka Rouhova, Hana Sehadova, Michal Sery, Miluse Hradilova, Peter Konik, Michal Zurovec

**Affiliations:** 1 Biology Centre of the Czech Academy of Sciences, Institute of Entomology, 37005 Ceske Budejovice, Czech Republic; rindom00@prf.jcu.cz (M.R.); lucie.kucerova@entu.cas.cz (L.K.); rouhol00@jcu.cz (L.R.); sehadova@entu.cas.cz (H.S.); kyklop@pf.jcu.cz (M.S.); 2 Faculty of Science, University of South Bohemia, 37005 Ceske Budejovice, Czech Republic; konik@prf.jcu.cz; 3 Institute of Molecular Genetics, Academy of Sciences of the Czech Republic, Videnska 1083, 142 20 Praha, Czech Republic; miluse.hradilova@img.cas.cz

**Keywords:** *Bena prasinana*, Bombycidae, Nolidae, phylogeny, transcriptomics, fibrohexamerins

## Abstract

Many lepidopteran larvae produce silk feeding shelters and cocoons to protect themselves and the developing pupa. As caterpillars evolved, the quality of the silk, shape of the cocoon, and techniques in forming and leaving the cocoon underwent a number of changes. The silk of *Pseudoips prasinana* has previously been studied using X-ray analysis and classified in the same category as that of *Bombyx mori*, suggesting that silks of both species have similar properties despite their considerable phylogenetic distance. In the present study, we examined *P. prasinana* silk using ‘omics’ technology, including silk gland RNA sequencing (RNA-seq) and a mass spectrometry-based proteomic analysis of cocoon proteins. We found that although the central repetitive amino acid sequences encoding crystalline domains of fibroin heavy chain molecules are almost identical in both species, the resulting fibers exhibit quite different mechanical properties. Our results suggest that these differences are most probably due to the higher content of fibrohexamerin and fibrohexamerin-like molecules in *P. prasinana* silk. Furthermore, we show that whilst *P. prasinana* cocoons are predominantly made of silk similar to that of other Lepidoptera, they also contain a second, minor silk type, which is present only at the escape valve.

## 1. Introduction

Many lepidopteran larvae produce silk serving in different forms as a protection of their larval and pupal stages. The silk consists of a fibrous hydrophobic core formed by fibroin molecules and a hydrophilic coating comprising adhesive proteins called sericins, as well as additional proteins with antimicrobial functions [1]. The amino acid composition of silk proteins greatly varies among Lepidoptera. The fibroin core consists of three fibroin molecules. Fibroin heavy chain (Fib-H), and fibroin light chain (Fib-L) are attached via a disulphide bond and their assembly is crucial for the intracellular transport of nascent silk [2]. Fibrohexamerin or fibroin P25 (Fhx/P25), contributes to the formation of the high molecular mass fibroin elementary units via hydrophobic interactions with the Fib-H and Fib-L complexes [3].

In the 1960s, X-ray structural analysis was performed on a number of natural materials, including silks from about 30 species belonging to different groups of Lepidoptera. According to the results, silks were divided into four classes depending on the composition of the so-called crystalline regions of Fib-H, formed by amino acid residues in the beta-conformation. These regions are responsible for the mechanical strength of the silk [4,5]. The crystalline regions are characterized by the intersheet packing distance of β-sheet crystallites and their specific sequence motifs [5,6] as follows: class I, intersheet packing distance of 9.3 Ǻ and alternating amino acids glycine and alanine or serine; class II, distance of 10 Ǻ and sequence ((Gly-Ala)_x_-Ala_y_)_n_; class III, distance of 10.6 Ǻ and polyalanine repeats; class IV (sometimes considered as atypical), distance of 15.0 Ǻ, with a low amount of alanine residues and a high proportion of serine, glutamic acid, and aspartic acid [5,6]. The sequences of several class III fibroins have been elucidated and its previously predicted polyalanine or alanine-serine regions were confirmed [7,8,9]. Furthermore, a recent study describing silk from *Eumeta* (*Clania*) *variegata*, a class II silk, contained ((Gly-Ala)_x_-Ala_y_)_n_ chains as predicted [10]. The silk of *P. prasinana* (originally belonging to the genus of *Bena*) has been placed in class I together with that of *Bombyx mori*; it has been suggested that the silks of both species have a similar structure, both containing long stretches of alternating glycine and alanine residues. The class I-X-ray structure of *B. mori* and *P. prasinana* silks is rare among Lepidoptera and has so far only been found in these two unrelated species (Figure S1).

**Figure 1:**
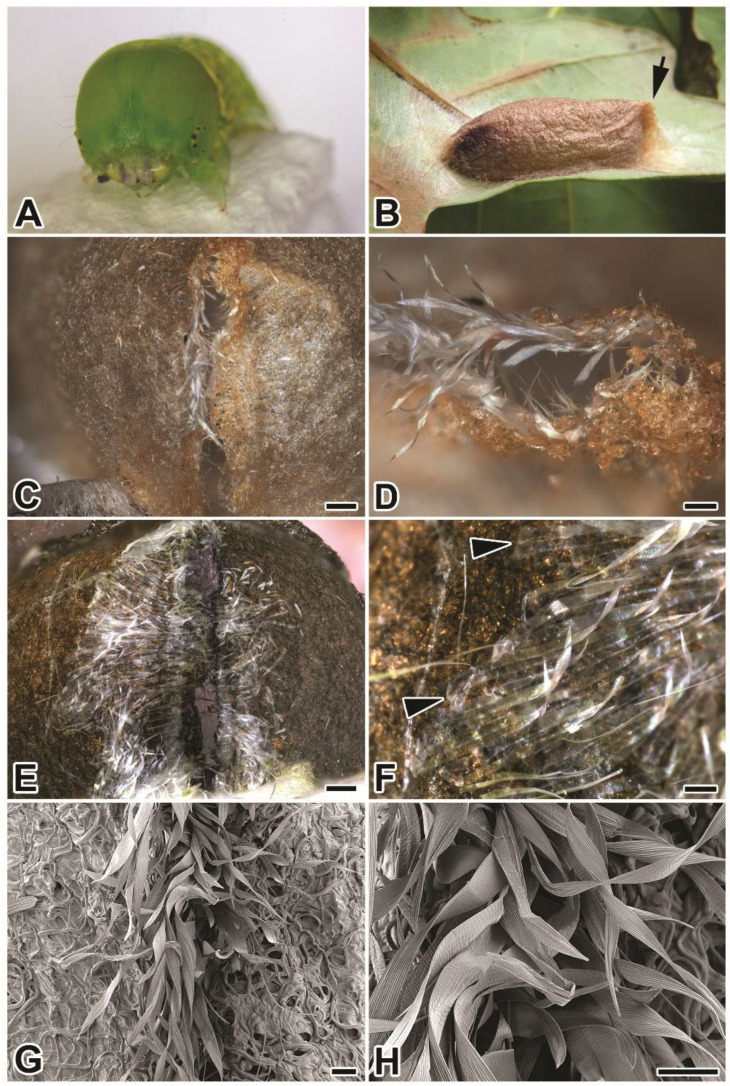
*Pseudoips prasinana* L. larva, cocoon and silk. (**A**) Last-instar larva. (**B**) Cocoon; arrow on the right points to the exit valve. (**C**,**D**) Outer and (**E**,**F**) inner side of the cocoon escape valve, examined using an optical microscope. (**F**) Arrowheads indicate the base of the newly detected silk type. (**G**,**H**) Scanning electron micrographs of the escape valve from the outside. *Scale-bars*: (**C**,**E**) 0.5 mm; (**D**,**F**) 0.2 mm; (**G**,**H**) 100 μm.

*P. prasinana* is a nocturnal bivoltine moth from the family Nolidae belonging to the superfamily Noctuoidea. It has a widespread distribution, occurring throughout the Palearctic region, and inhabits various types of forest habitat, where it feeds mainly on beech and oak leaves. Fully grown larvae are green and approximately 4 cm long (Figure 1A) and adults have a wingspan 30–40 mm. Pupation takes place in a boat-shaped parchment-like cocoon (Figure 1B) on the underside of the leaf [11,12].

Since the studies of the 1960s, many new methodological approaches have been developed, providing the opportunity to study physiology in much more detail, as well as enabling systematic comparisons of biological systems that were not previously possible. ‘Omics’ technologies have revolutionized silk research, and are able to generate massive datasets that cover transcribed genes and proteins of entire tissues, a significant improvement over the traditional time-consuming isolation and analysis of single molecules [13]. In the present study, we analyzed the protein composition of *P. prasinana* cocoons and detected and annotated 136 proteins, included the proteins of the fibroin fraction as well as four sericins, seroin, and protease inhibitors. Characterizing *P. prasinana* silk and comparing it with that of *B. mori* will help to understand the mechanism of silk protein changes during evolution as well as elicit the function of individual silk components. Our results show that fibrohexamerin and fibrohexamerin-like genes expanded in *P. prasinana*, and therefore likely cause the difference in mechanical properties between *P. prasinana* and *B. mori* silks.

## 2. Results

### 2.1. Compact Structure of P. prasinana Cocoons

*Pseudoipsprasinana* cocoons are approximately 2.5 cm long and have the appearance of parchment; they are more compact and thinner than those of *B. mori*. They also differ from *B. mori* cocoons by having a boat shape with a wider anterior part ending in a pointed thinned edge. The edge structure, which resembles a seam, serves as an escape valve for the emerging adult (Figure 1B–H).

**Figure 2:**
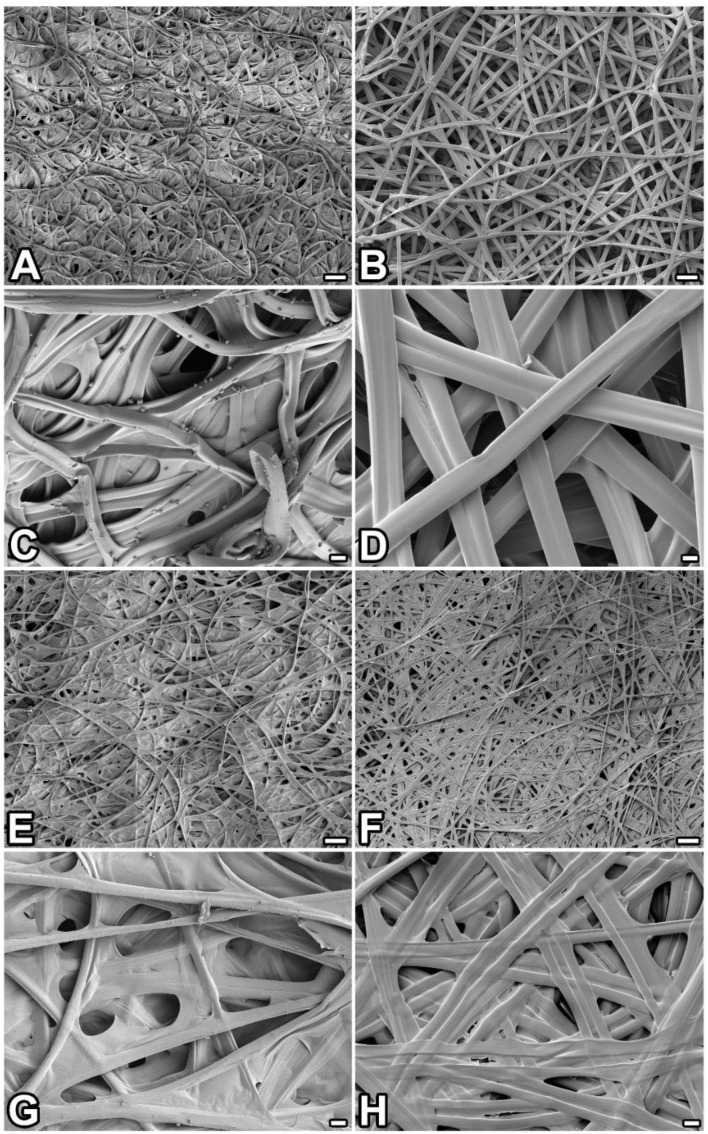
Comparison of *P. prasinana* and *B. mori* silks using scanning electron microscopy. (**A–D**) Outer and (**E**–**H**) inner sides of the cocoon. Images on the left (**A**,**C**,**E**,**G**) show that of *P. prasinana* whilst images on the right (**B**,**D**,**F**,**H**) are from *B. mori. Scale-bars*: (**A**,**B**,**E**,**F**) 100 μm; (**C**,**D**,**G**,**H**) 10 μm.

Further examination using light and scanning electron microscopy revealed that *P. prasinana* silk filaments mostly form arched structures, indicating that the larva performs semi-circular movements with its head during cocoon spinning (Figure 2A–H). In contrast, the silk architecture at the seam region appears different and delimits the escape valve opening. The opening is covered from the inner side of the cocoon by transparent ribbon-like fibers. These ribbons are colorless, flat, and wider than the regular silk fibers which make up the cocoon (the width of the ribbon silk is 58–69 μm, while the main cocoon silk has a diameter of 9–13 μm). Their distal ends are glued to the inner side of the cocoon around the escape valve, while the proximal part is loose and covers the opening. As shown in Figure 1C,D,G,H, the ribbons are partially pushed outwards when the adult moth passes through.

**Figure 3:**
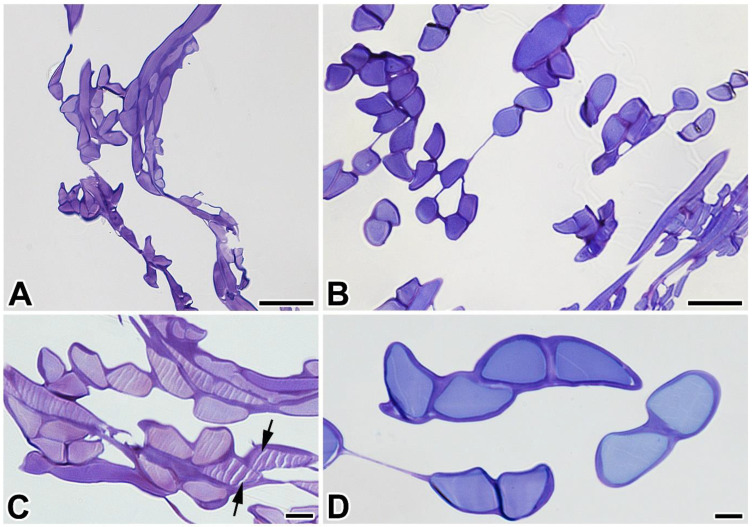
Light microscopy of toluidine-blue stained semi-thin sections of (**A**,**C**) *P. prasinana* and (**B**,**D**) *B. mori* cocoons. The inner fibroin (lighter) and outer sericin (darker) layers can be distinguished. Arrowheads in the figure C point to parallel cracks that could be artifacts related to knife marks. *Scale-bars*: (**A**,**B**) 20 μm; (**C**,**D**) 5 μm.

To find out more about the differences between *P. prasinana* and *B. mori* silks, we prepared semi-thin sections for light microscopy. As shown in Figure 3A–D, *P. prasinana* silk fibers are thinner than that of *B. mori* (9–13 μm thick compared to 10–15 μm, respectively). The *P. prasinana* cocoon seems to contain fewer layers and a higher proportion of glue components (Figure 3C). It is easy to distinguish the less stainable fibroin core from the more stainable sericin cover in the sections (Figure 3C,D). The fibroin core of *P. prasinana* silk in the figure has distinct parallel cracks that could be artifacts related to knife marks or fibroin core shrinkage during dehydration.

### 2.2. Mechanical Strength Measurement

**Figure 4:**
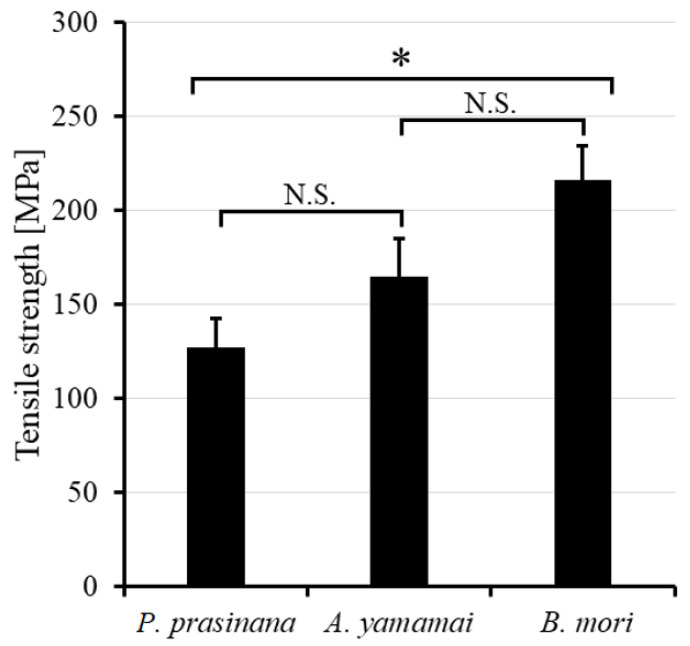
Tensile properties of *P. prasinana*, *B. mori* and *A. yamamai* silk fibers tested after degumming and drying for 2 h. At least four tests were carried out for each fiber type. Error bars indicate standard error of the mean. Asterisk indicates a statistically significant difference (*p* < 0.05), N.S. indicates not significant (*p* > 0.05).

To compare the mechanical strength of *P. prasinana* silk with that of other silks, we used three moth species: *P. prasinana*, *B. mori*, and *A. yamamai.* The tensile strength of *B. mori* and *A. yamamai* silk was measured earlier, and *A. yamamai* fibers showed 22% lower strength compared to *B. mori* fibers. As it was difficult to obtain fibers from the *P. prasinana* cocoon, we released individual fiber pairs by degumming. The samples were heated in glass beakers in distilled water for 15 min at 95 °C, then the solution was allowed to cool to 50 °C and the process was repeated twice. First, we tested the tensile strength of fibers which had been air-dried for 24 h. However, the *P. prasinana* fibers were quite brittle and it was difficult to get reproducible data. We therefore shortened the drying time to 2 h, which reduced the fiber fragility. The forces required to break the individual fibers are shown in Figure 4. The results show that *P. prasinana* fibers are 42% and *A. yamamai* fibers are 23% weaker than *B. mori* silk.

### 2.3. Transcriptome Construction, Proteomic Analysis

In order to characterize *P. prasinana* silk at the molecular level, we prepared a silk gland-specific cDNA library. We sequenced more than 16 million reads, which were assembled into 21,000 contigs. We then used local BLAST in BioEdit (v.7.2) software to search for Fib-H, Fib-L, Fhx/P25, and sericin homologs. To analyze silk components, we performed a proteomic analysis of the cocoon. First, the cocoon was dissolved in urea, trypsinized, and then analyzed by chromatography coupled to tandem mass spectrometry, as described in Materials and Methods. The resulting peptide spectra were identified using the protein database derived from our transcriptome.

A proteomic analysis of the *P. prasinana* silk revealed 136 protein components, which were annotated using searches against the NCBI non-redundant database (Supplementary Materials Table S1). These proteins included structural silk components containing signal peptides, together with non-secretory cellular proteins (ribosomal and cytoskeletal proteins, enzymes) and 32 incomplete unidentified short protein fragments. Out of the 136 components, 59 (43.3 %) contained signal peptides, 33 had homologs in other moth species (which were not associated with silk, i.e., enzymes, hemolymph proteins), 26 mostly corresponded to proteins with homology in other moth silks (see Supplementary Materials Table S2) including Fib-H, Fib-L, 14 were fibrohexamerins and fibrohexamerin-like proteins, two were sericins (two more putative sericins were identified at the transcriptomic level), one was a seroin, and two were zonadhesin- like proteins. The remaining five proteins were completely unknown.

As shown in Supplementary Materials Table S2, most *P. prasinana* silk components are hydrophilic, except for Fib-L. The most hydrophilic proteins are sericins. The sequences of large proteins containing repetitive regions are incomplete due to difficulty in assembling. Some silk proteins showed characteristic proportions of amino acid residues. The most abundant amino acid in Fib-H is Gly, followed by Ala and Ser, while Fib-L is the richest in Ala, Leu, and Val. In particular, Leu was also found to be very common in all fibrohexamerin-like proteins. Sericins usually contain a high proportion of Ser, whilst zonadhesin-like proteins are rich in Cys (Supplementary Materials Table S2).

### 2.4. Major Silk Structural Proteins and Their Phylogenetic Relationships

In order to identify evolutionary relationships among silk proteins, we performed a phylogenetic analysis and reconstructed dendrograms based on the Maximum Likelihood (ML) method. The sequence alignments of the central repetitive fibroin-H sequence between *P. prasinana* and *B. mori* showed close similarity (more than 72% amino acid identity in the central repeat region of 250 amino acid residues). *P. prasinana* Fib-H contained long stretches of GAGA motifs, which are also typical for the *B. mori* crystalline region (Supplementary Materials Figure S2). However, unlike silkworm Fib-H, *P. prasinana* Fib-H did not contain amorphous regions and, conversely, contained slightly more serine and tryptophan residues dispersed within the repeats, making the molecule less hydrophobic (Supplementary Materials Table S3).

**Figure 5:**
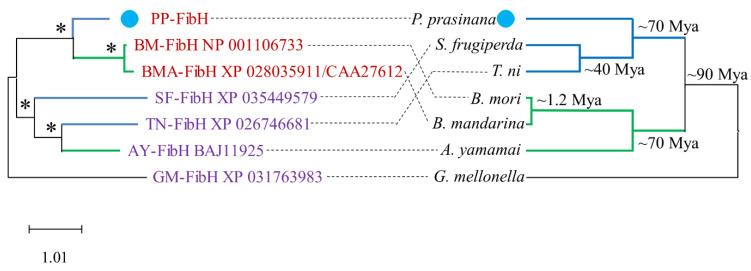
Comparison of available Fib-H protein sequences (left). The *P. prasinana* (PP) Fib-H sequence (blue dot) is more similar to that of *B. mori* (BM) and *B. mandarina* (BMA), these three sequences form a single well-supported cluster based on the similar structure of their crystal region (Class I sequences are marked by red text). Fib-H sequence from *Antheraea yamamai* (AY), another representative of superfamily Bombycoidea, forms one well supported cluster with two sequences from Noctuoidea (*Spodoptera frugiperda*, SF, and *Trichoplusia ni*, TN). Based on crystal region structure they all belong to Class III (text marked by violet), including the outgroup sequence from the pyralid moth, *Galleria mellonella* (GM). Corresponding species tree with time divergence of nodes is shown on the right. All species from Bombycoidea superfamily have branches highlighted in green and all species from Noctuoidea superfamily have branches highlighted in blue (Mya = million years ago). Fib-H tree (ML) exhibits full resolution and good statistical support. Branches with statistical support (aBayes values) higher than 70 are marked with asterisks.

The phylogenetic analysis of Fib-H protein sequences assigned *P. prasinana* Fib-H to the same cluster as *B. mori* due to the high similarity in repeated regions. Thus, the Fib-H gene tree does not follow the species tree and rather reflects adaptive changes and convergent evolution (Figure 5). In contrast, the developmental history of Fib-L shows no peculiarity and its cladogram corresponds to that of the species. *Pseudoips* *prasinana* Fib-L shares 60% amino acid identity with *B. mori* Fib-L (Supplementary Materials Figure S3).

**Figure 6:**
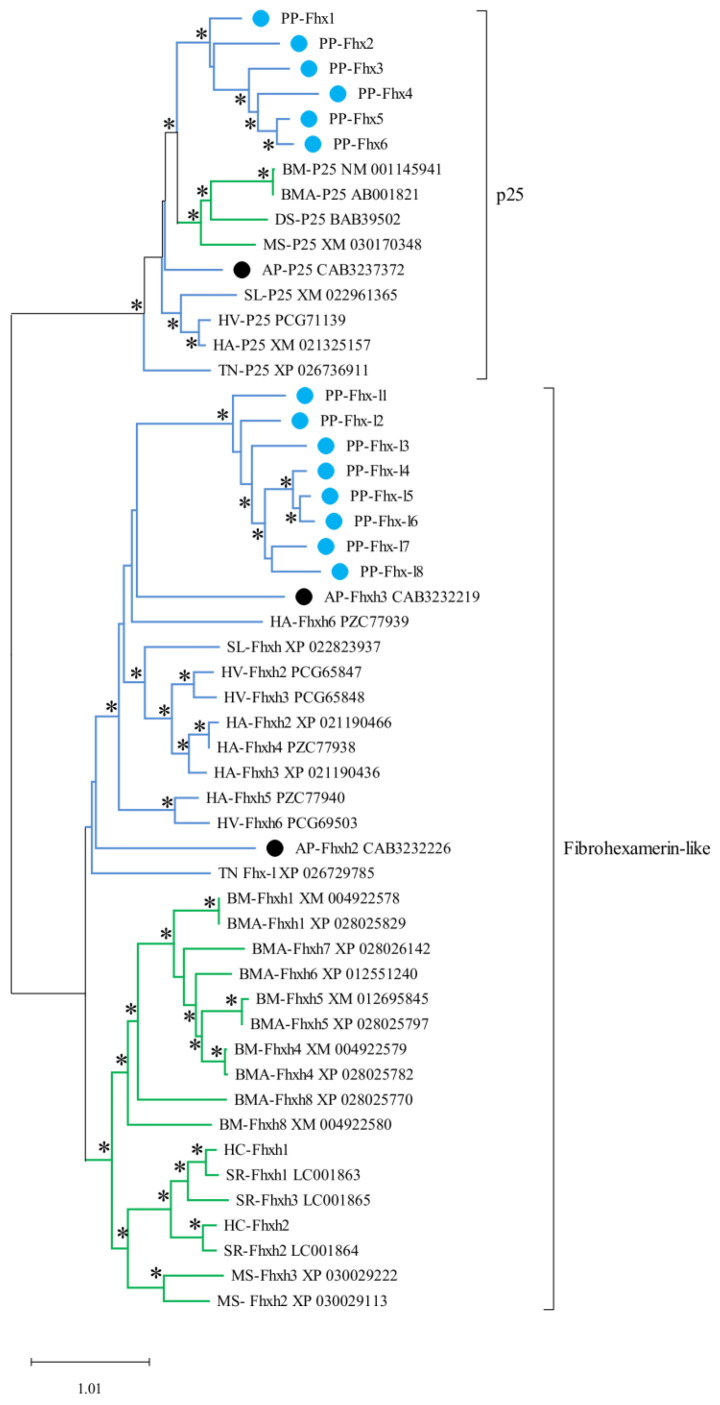
Expansion of fibrohexamerins and fibrohexamerin-like proteins in *P. prasinana*. The dendrogram also contains sequences from several members of the superfamily Bombycoidea (shown with green lines; *B. mori*, BM; *B. mandarina*, BMA; *Manduca sexta*, MS; *Dendrolimus spectabilis*, DS; *Samia ricini*, SR; *Hyalophora cecropia*, HC) and Noctuoidea (shown with blue lines; *S. litura*, SL; *Heliothis virescens*, HV; *Helicoverpa armigera*, HA; *T. ni*, TN). *Pseudoips* *prasinana* (PP) sequences are highlighted by blue dots whilst sequences from its closest studied relative, *A. plantaginis* (AP), are highlighted by black dots. The maximum-likelihood method was used to reconstruct the phylogeny. Branches with statistical support (Bayes values) higher than 70 are marked with asterisks.

Interestingly, *P. prasinana* silk contains a dramatically higher number of fibrohexamerins and fibrohexamerin-like proteins compared to silks of other Lepidoptera. Their molecular weight ranges from 21 to 28 kDa and they tend to be hydrophilic. The phylogenetic analysis revealed that fibrohexamerin proteins form two main groups: bona fide fibrohexamerins (close homologs of *B. mori* P25), and fibrohexamerin-like proteins (Figure 6). The bona fide fibrohexamerins (P25 proteins) in most other species, including *B. mori* as well as some studied noctuoid species, are encoded by a single copy gene [14]. In contrast, we found six bona fide Fhx/P25 paralogs in *P. prasinana*, which is unique among Lepidoptera. In addition, the duplication also occurred in the other group of fibrohexamerin-like proteins. *Pseudoips* *prasinana* thus has more fibrohexamerin-like homologs than *B. mori* (8 vs. 6) or indeed any other moth studied to date. In addition, the branching of the cladogram showed two species-specific clusters in *P. prasinana*, thus supporting the idea that both fibrohexamerin groups expanded quite recently.

We found only two sericins, Src1A and Src1B, in the *P. prasinana* cocoon by proteomic methods. These seem to be orthologs of *B. mori* sericin 1, which are characterized by two conserved cysteine residues of the CXCX motif near the C-termini. We also searched our transcriptome by running a local BLAST search using known sequences of sericins from other moths. We detected at least two other putative sericin proteins, named Src2 and Src3, which contained putative signal peptides, repetitive sequences in the central part of the molecule and a high percentage of serine residues (Supplementary Materials Table S2). The phylogenetic analysis confirmed that these two additional sericins are related to sericin 2 from *B. mori* (Supplementary Materials Figure S4).

## 3. Discussion

Lepidopteran caterpillars produce silk from a pair of labial glands in the form of two filaments. Both filaments are aligned and sealed together into a fiber by sericin adhesives during its passage through the common spinneret [15]. The diameter of the spun-out silk increases with age and developmental stage. The tensile strength of silk materials depends mainly on its crystallites, highly organized β-sheet structures in Fib-H [16,17]. The amino acid sequences of the central repetitive regions of *P. prasinana* and *B. mori* Fib-H forming the crystallites are almost identical, even though the moth species are not closely related. The estimated separation of Noctuoidea and Bombycoidea occurred during the Cretaceous period approximately 70 million years ago. Both fibroins are of an unusual type, characterized by high glycine content, followed by alanine, serine, and tyrosine [4]. The glycine residues alternate with alanine in the crystalline regions, thus allowing a special arrangement with the small intersheet packing distance of 9.3 Ǻ characteristic for group I fibroins or group I silks [5].

Our measurements of the tensile strength of *B. mori* and *A. yamamai* fibers showed significantly lower values compared to that of previous studies [18,19]. This might be due to the relatively harsh degumming methodology used in our experiments, which may have affected their mechanical properties [20]. Importantly, the difference in relative tensile strength was significant, with *P. prasinana* fibers much weaker than that of *B. mori*. Class I silks such as these are supposed to be stiff in comparison to other silks based on measurements in *B. mori* [19]. Overall, *P. prasinana* fibers did not show any obvious structural difference from that of *B. mori*, which would explain its low tensile strength. We hypothesize that the observed low tensile strength is related to the high proportion of Fhx/P25 and Fhx-like proteins in the silk core. Alternatively, *P. prasinana* silk might be more sensitive to the degumming process than that of *B. mori* and *A. yamamai.*

The role of Fhx/P25 and Fhx-like proteins in the silk core is not clear. Studies on *B. mori* Fhx/P25 suggested that they are responsible for the efficient secretion of fibroin complexes from posterior silk gland (PSG) cells into the lumen, as well as maintaining the solubility of fibroin complexes during luminal transport [21]. It was previously shown in *B. mori* that Fib-H and -L chains, as well as Fhx/P25, were all present in the endoplasmic reticulum (ER) fraction of PSG cells and the remaining Fhx/P25 which was not assembled into the fibroin elementary unit stayed in the ER [22]. Interestingly, previous experiments with transgenic silkworms carrying a truncated Fib-H variant chimeric Fib-H-GFP suggested that its C-terminal region interacting with Fib-L was not needed for the secretion of the chimeric protein. However, such truncated Fib-H containing the intact N-terminal Fib-H region could still interact with Fhx/P25 [23]. Since all 14 Fhx/P25 and Fhx-like proteins were detected in the *P. prasinana* cocoon, they may all interact with the Fib-H or Fib-H-Fib-L complexes as a part of the silk core.

Interestingly, the expression of other Fhx-like proteins in *B. mori* (e.g., XM_004922580 (BMSK0008085) or XM_004922578 (BMSK0008085)) predominantly occur outside of the silk glands (e.g., in the larval trachea or epidermis) [24]. Fhx-like genes in a more primitive lepidopteran species, *Tineola bisseliella Fhx1* and *Fhx2*, are expressed predominantly in the middle silk gland (MSG) [14]. Such differences in tissue specificity suggest that at least some of the Fhx/P25 and Fhx-like proteins may play roles other than interacting with fibroin complexes within the PSG.

The beta sheet structures in the Fib-H molecule are responsible for fiber strength and, due to the limited number of possible conformations, the same structures can appear in distant species (Figure S1) [4]. The presence of beta sheets in the Fib-H molecule may sometimes represent a significant structural limitation to the amino acid sequence, making adaptive changes difficult. It has previously been suggested that the extraordinary mechanical properties of silk from some species may be incidental and exceed the functional requirements of the species [25]. A high proportion of Fhx proteins in the silk might change the mechanical properties of the entire complex. A high proportion of Fhx protein caused by gene multiplication might be an adaptation to the desired lower silk strength. Clade-specific gene duplications or gene losses seem to be a common strategy for improving the silk properties of Lepidoptera. For example, sericin gene multiplication was observed in *Galleria mellonella* [26] and the loss of Fib-L and Fhx/P25 was observed in the saturniid family [27].

The cocoon of *P. prasinana* seems to be unique in that it contains two distinct fibers of different morphology, both in structure and diameter (Figure 1A–H). The major cocoon silk is quite similar to that of other Lepidoptera: the fibers are uniform, with a diameter of 9–13 μm. However, a second type of fiber exists in a much lower quantity. It has a ribbon-like morphology and is localized at the inner anterior side of the cocoon around the escape valve. Such ribbon fibers have not been observed in any other Lepidoptera to date. Our results show that it might be specific for *P. prasinana*, serving as a fine flexible protective inner cover for the escape valve. The ribbons are flat, transparent, and have a width of 58–69 μm. The ribbon fibers are attached distally at the inner cocoon wall and become loose as they extend across the escape valve by forming fringe curtains (Figure 1). The localization of ribbon silk suggests that it was added at the end of spinning. The different morphology of these ribbon-like fibers indicates that they may not be products of the labial silk glands, but rather some other larval secretory gland (possibly maxillary glands). *Pseudoips* *prasinana* cocoons may therefore contain products of two distinct secretory glands. A detailed analysis is needed to clarify the composition and origin of the ‘ribbon silk’.

We describe herein a number of novel silk proteins from the noctuoid moth *P. prasinana*, which has an X-ray silk structure very similar to that of *B. mori*. We show that, despite the close sequence similarity between Fib-H molecules of *P. prasinana* and *B. mori*, the resulting fibers exhibit quite different mechanical properties. We observed multiplication of Fhx/P25 and Fhx-like genes, which may be responsible for the lower tensile strength of *P. prasinana* silk. Moreover, *P. prasinana* cocoon contains two distinct fibers, which may represent products of two different larval secretory glands. This study will improve our understanding the role and evolution of silk components.

## 4. Materials and Methods

### 4.1. Insects

Two female *P. prasinana* were collected during a light trapping program in June 2017 in Bonyhad (Hungary). The eggs hatched 14 days after laying, and the larvae were fed with oak leaves for 8 weeks. Larvae of the *B. mori* polyvoltine line N4 were obtained from the Institute of Zoology, Bratislava (Slovakia) and kept at low density on an artificial diet (Nihon Nosan Corporation, Yokohama, Japan) or mulberry leaves at 25 °C. The breeding material of *Antheraea yamamai* was purchased from Worldwide Butterflies (https://www.wwb.co.uk).

### 4.2. Histology and Scanning Electron Microscopy

Semi-thin cocoon sections were prepared as previously described [26]. Briefly, pieces of cocoon were prepared in phosphate buffered saline (PBS) and fixed in 2.5% glutaraldehyde/PBS overnight at 4 °C. The samples were washed and sequentially dehydrated by adding acetone to PBS at increasing concentrations (30%, 50%, 70%, 80%, 90%, 95%, and 100%), with 15 min incubation for each stage. The dehydrated samples were embedded in Epon resin and left in undiluted resin for 24 h at room temperature and for 48 h at 62 °C. Semi-thin sections were cut with a glass knife and stained with toluidine blue and viewed under a light microscope. Macrophotographs were taken using a D5600 digital camera (Nikon) and SZX16 stereomicroscope (Olympus). The final images were created by merging several Z stacks.

Scanning electron microscopy was performed as described in a previous study by Zurovec et al. [28]. Cocoon pieces were glued to aluminium holders, sputter-coated with gold, and analyzed with a JSM-7401F scanning electron microscope (Jeol).

### 4.3. Measurement of Silk Fiber Mechanical Strength

The cocoons of *P. prasinana*, *B. mori* and *A. yamamai* were soaked in hot water (3 × 15 min) to dissolve sericins and thus release fibers from the cocoon wall. Silk fibers were collected from the outer surface of the cocoon and were then air-dried at room temperature, either overnight or for 2 h. Fiber diameter was measured under a BX51 light microscope (Olympus) equipped with Nomarski optics. For mechanical testing, fibers approximately 2 cm long were individually attached to the mounting surfaces of the testing apparatus, which measured the force of lifting a weight on miniature scales. The diameter of the spun fiber was measured at 8 locations along its length, with the cross-sectional area assumed as circular. The fiber was pulled at a constant speed of 0.5 mm/s with a slowly rotating motor and the force to which it was exposed was read on a computer as a reduction of the original weight load. The measurements were terminated when the fiber was pulled apart. The tensile tests were statically analyzed by one-way analysis of variance (Supplementary Materials Table S4).

### 4.4. Transcriptome Preparation and Analysis

RNA isolation, synthesis of cDNA libraries and RNA sequencing were performed as previously described [28]. Total RNA from the dissected larval silk glands of *P. prasinana* was isolated using Trizol reagent (Life Technologies, Carlsbad, CA, USA) and used to prepare cDNA libraries for the Illumina sequencing platform. rRNA was removed using “A RiboMinus Eukaryote Kit for RNA-Seq” (Ambion, Austin, TX, USA). poly-A mRNA was enriched using a Dynabeads Oligo (dT)_25_ mRNA Purification Kit (Thermo Fisher Scientific, Waltham, MA, USA), and the cDNA library was prepared with a NEXTflex Rapid RNA-Seq Kit (Bioo Scientific, Austin, TX, USA). Sequencing was performed using a MiSeq instrument (Illumina, San Diego, CA, USA), producing sequences in the 2 × 150 nt pair-end format. A total of 1.6 × 10^7^ reads were assembled *de novo* using Trinity software (v.2.9.1) on the Galaxy platform [29] set on the default options and a minimum allowed length of 200 bp. The transcriptome database served for manual searches of homologs of known silk proteins from other species by local BLAST in BioEdit (v.7.2) software [30] and also as a backbone for the identification of tryptic peptides in MS spectrometry. The nucleotide sequences were uploaded to NCBI GenBank under the codes MW373748–MW373775.

### 4.5. Protein Extraction and Identification of Protein Fragments by Mass Spectrometry

About 10 mg of *P. prasinana* silk was dissolved in 8 M urea and further processed using SP3 beads, according to Hughes et al. [31]. Briefly, after washing, the samples were trypsinized overnight, acidified with formic acid to 1% final concentration, and peptides were desalted using C18 disks (Empore) according to Rappsilber et al. [32]. The samples for LC/MS analysis were processed as described previously [33]. Max Quant and its search engine Andromeda [34] were used to search MS/MS spectra against the *P. prasinana* database on the basis of RNA-Seq data. Further data analysis was performed using Perseus software (v.1.5.2.4) [35].

### 4.6. Phylogenetic Analysis

Protein sequences were aligned with the MUSCLE algorithm [36]. For each protein alignment the best model (WAG + G+F) was chosen by Smart Model Selection (SMS) [37] according to the lowest Bayesian information criterion (BIC) scores. The resulting aligned matrix was manually trimmed. Phylogenetic maximum-likelihood (ML) analysis was carried out using the original algorithm, Nearest Neighbor Interchange (NNI), in PhyML (v.3.0) [38]. MEGA-X software [39] was used to graphically adjust the phylogenetic trees. Branch support (aBayes values) higher than 70 is marked on the phylogenetic trees with asterisks (*).

## Data Availability

All datasets are provided within the manuscript itself or the electronic Supplementary Material.

## Supplementary Materials

The following are available online at https://www.mdpi.com/article/10.3390/ijms22158246/s1.
