# Supplementary material for: Comparison of Silks from Pseudoips prasinana and Bombyx mori Shows Molecular Convergence in Fibroin Heavy Chains but Large Differences in Other Silk Components
Source: Int J Mol Sci. 2021 Jul 31;22(15):8246. doi: 10.3390/ijms22158246 (PMC8347419; doi:10.3390/ijms22158246)
Supplement: Supplementary file 1 [file ijms-22-08246-s001.zip › Table S4.pdf]

Table S4. Statistical analysis of the silk tensile strengths of studied moth species

ANOVA

Group	No.	Sum	Mean	Variance
Pseudoips prasinana	11	1 402	127	2 888
Antheraea yamamai	6	991	165	2 476
Bombyx mori	4	864	216	1 375

Table X.

ANOVA

Source of variation	SS	df	MS	F	*p* -value	F crit
Between groups	23 885	2	11 942	4.74	0.022	3.55
Within Groups	45 384	18	2 521			
Total	69 269	20				

Table X.

Post-hoc test	t (*p* -value)	Sign. (Bonferroni ad.)
P . prasinana vs *A . yamamai*	0.175	FALSE
A . yamamai vs *B . mori*	0.101	FALSE
P . prasinana vs *B . mori*	0.007	TRUE
