# Supplementary material for: Comparison of Silks from Pseudoips prasinana and Bombyx mori Shows Molecular Convergence in Fibroin Heavy Chains but Large Differences in Other Silk Components
Source: Int J Mol Sci. 2021 Jul 31;22(15):8246. doi: 10.3390/ijms22158246 (PMC8347419; doi:10.3390/ijms22158246)
Supplement: Supplementary file 1 [file ijms-22-08246-s001.zip › Table S3.pdf]

Table S3. A comparison of parameters and the most abundant amino acids of Fib-H of P. prasinana and B. mori calculated by ProtParam (https://web.expasy.org/protparam).

**Fibroin parameters**	***P . prasinana***	***B . mori***
Hydrophobicity (GRAVY)	-0.078	0.243
Theoretical pI	5.07	3.73
Glycine	40.7%	47.4%
Alanine	24.7%	31.2%
Serine	15.9%	12%
Tyrosine	7.6%	5.2%
