# Supplementary material for: Comparison of Silks from Pseudoips prasinana and Bombyx mori Shows Molecular Convergence in Fibroin Heavy Chains but Large Differences in Other Silk Components
Source: Int J Mol Sci. 2021 Jul 31;22(15):8246. doi: 10.3390/ijms22158246 (PMC8347419; doi:10.3390/ijms22158246)
Supplement: Supplementary file 1 [file ijms-22-08246-s001.zip › Table S2.pdf]

Table S2. Gene products found in the *P. prasinana* silk. List of the GenBank accession numbers, their sizes. Protein parameters including hydrophobicity and quantity (%) of three most frequent amino acids were determined by ProtParam (https://web.expasy.org/protparam).

Name	Symbol	GenBank ID	Size (kDa)	Hydrophobicity	1st AA (%)	2nd AA (%)	3rd AA (%)
Fibroin heavy chain	Pp-FibH	MW373748	incomplete	-0.078	G (31,2)	A (19,1)	S (17,1)
Fibroin light chain	Pp-FibL	MW373749	27514.28	0.233	A (16,9)	L (10,5)	V (10,2)
Sericin 1A	Pp-Src1A	MW373764	incomplete	-1.068	S (29,3)	G (15,2)	N (9,9)
Sericin 1B	Pp-Src1B	MW373765	incomplete	-1.101	S (27,7)	G (14,2)	D (8,2)
Sericin 3	Pp-Src3	MW373766	incomplete	-1.182	S (30,7)	G (11,6)	T (8,5)
Sericin 2	Pp-Src4	MW373767	incomplete	-1.035	S (20,0)	G (13,1)	N (11,2)
Fibrohexamerin 6	Pp-Fhx6	MW373750	26851.74	0.009	L (10,6)	R (8,1)	A (7,2)
Fibrohexamerin-like 5	Pp-Fhx-l5	MW373751	25685.28	-0.042	L (11,4)	Y (7,9)	V (7,4)
Fibrohexamerin-like 2	Pp-Fhx-l2	MW373752	26158.12	-0.08	L (10,1)	F,T,V (7,0)	K,S,Y (6,6)
Fibrohexamerin-like 1	Pp-Fhx-l1	MW373753	25744.59	0.005	L (8,7)	G, V (7,8)	S (7,0)
Fibrohexamerin-like 3	Pp-Fhx-l3	MW373754	27199.23	-0.257	V (8,5)	Y (8,1)	L (7,7)
Fibrohexamerin 3	Pp-Fhx3	MW373755	27645.71	-0.223	L (11,5)	R (11,1)	Y (8,5)
Fibrohexamerin-like 6	Pp-Fhx-l6	MW373756	21105.98	-0.228	L (8,6)	Y (8,1)	N, V (7,6)
Fibrohexamerin-like 4	Pp-Fhx-l4	MW373757	26295.91	-0.22	L (11,4)	Y, V (8,3)	N (7,9)
Fibrohexamerin 4	Pp-Fhx4	MW373758	incomplete	-0.164	R, L (10,5)	V (9,1)	S (8,2)
Fibrohexamerin 5	Pp-Fhx5	MW373759	27277.09	-0.193	L (11,0)	R (10,2)	Y (8,1)
Fibrohexamerin 1	Pp-Fhx1	MW373760	25850.81	0.019	L (12,4)	R,V (8,0)	N,I (7,5)
Fibrohexamerin 2	Pp-Fhx2	MW373761	25725.58	-0.062	I,L (8,0)	R,F,S,V (6,6)	D (6,2)
Fibrohexamerin-like 8	Pp-Fhx-l8	MW373762	27848.15	-0.063	L (10,5)	V (7,7)	S (7,3)
Fibrohexamerin-like 7	Pp-Fhx-l7	MW373763	27936	-0.292	L (8,3)	Y,V (7,9)	I,S (6,6)
Zonadhesin-like 1	Pp-Zon1	MW373768	166098.08	-0.432	C (15,7)	G (9,0)	P (8,9)
Zonadhesin-like 2	Pp-Zon2	MW373769	59825.97	-0.433	C (13,5)	P (11,3)	G (8,5)
Seroin 1-like	Pp-Srn1	MW373775	26666.2	-0.464	F (11,1)	P (10,7)	G,T (9,4)
D rich short acidic peptide	Pp-Dsap	MW373771	12964.18	-0.552	D (11,9)	E,G (9,3)	V (8,5)
E rich short acidic peptide	Pp-Esap	MW373772	10453.77	-0.567	E (13,3)	A,Y (10,0)	L (8,9)
Predicted conserved lepidopteran protein phosphatase inhibitor	Pp-Clpi	MW373773	21724.9	-0.73	N (10,9)	Q (9,9)	E (8,9)
Histidine acid phosphatase Acph-1-like	Pp-Acph1	MW373774	44223.73	-0.262	L (13,4)	E (7,5)	G (7,2)
ZP domain containing secretory protein	Pp-Zpsp	MW373770	incomplete	-0.106	V (9,4)	L (9,0)	A (8,7)
