# Supplementary figures and images for: Comparison of Silks from Pseudoips prasinana and Bombyx mori Shows Molecular Convergence in Fibroin Heavy Chains but Large Differences in Other Silk Components
Source: Int J Mol Sci. 2021 Jul 31;22(15):8246. doi: 10.3390/ijms22158246 (PMC8347419; doi:10.3390/ijms22158246)

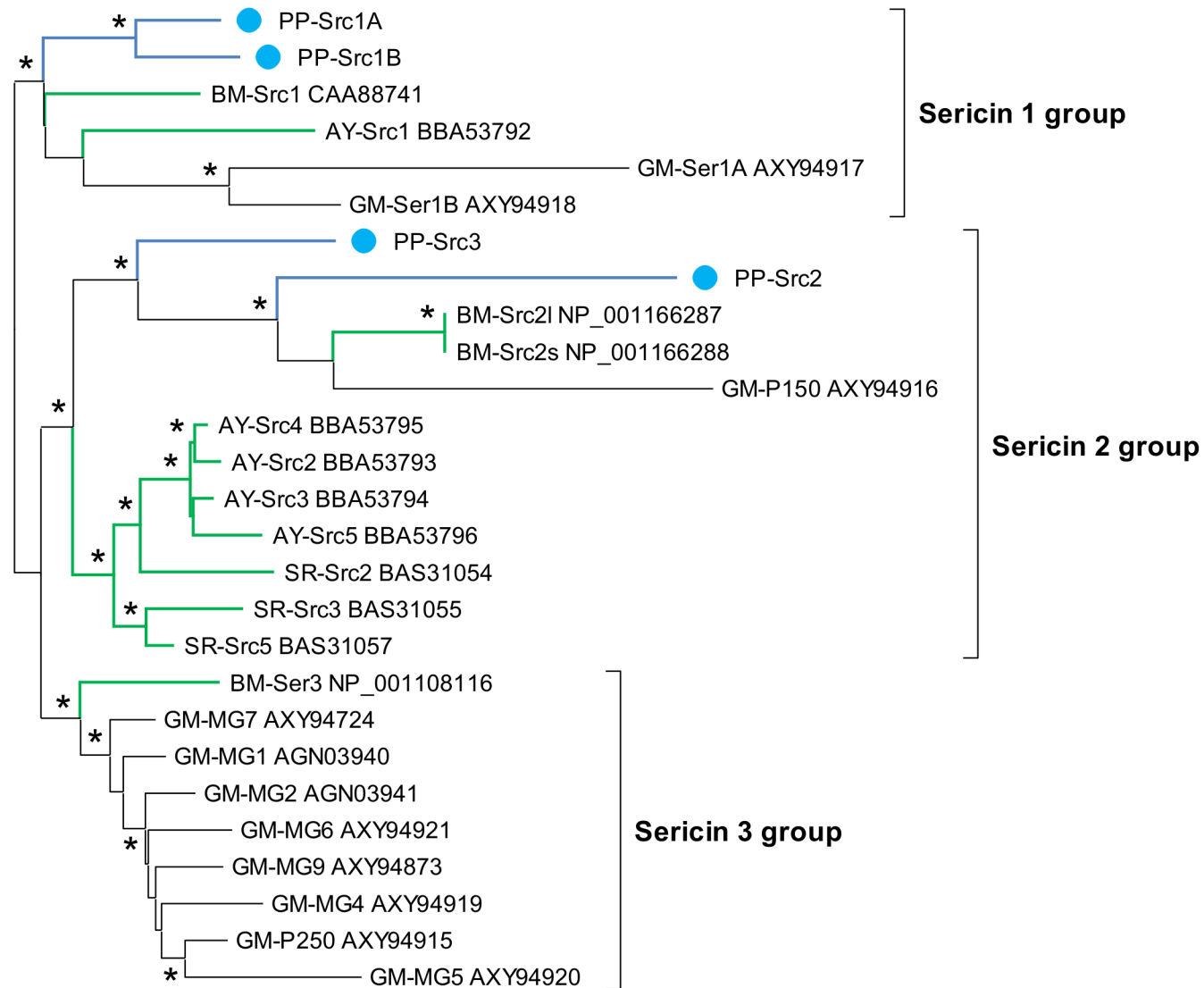

2.1

Supplement: Supplementary file 1 [file ijms-22-08246-s001.zip › Figure S4.pdf]

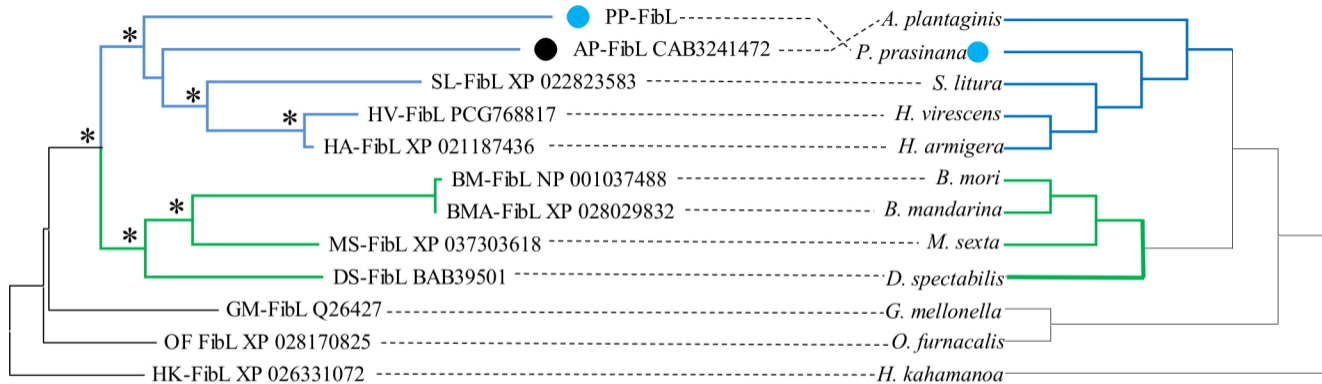

0.5

Supplement: Supplementary file 1 [file ijms-22-08246-s001.zip › Figure S3.pdf]

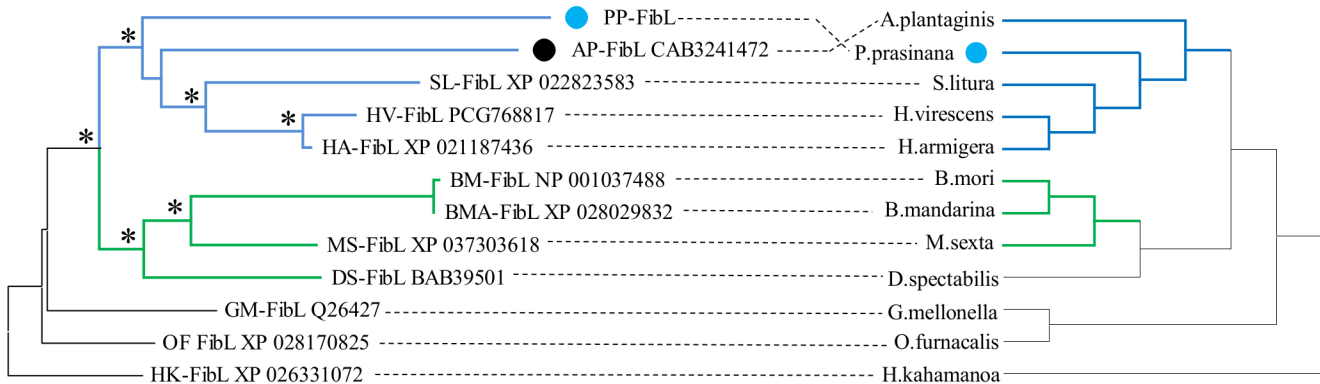

0.5

Supplement: Supplementary file 1 [file ijms-22-08246-s001.zip › Figure S2.pdf]

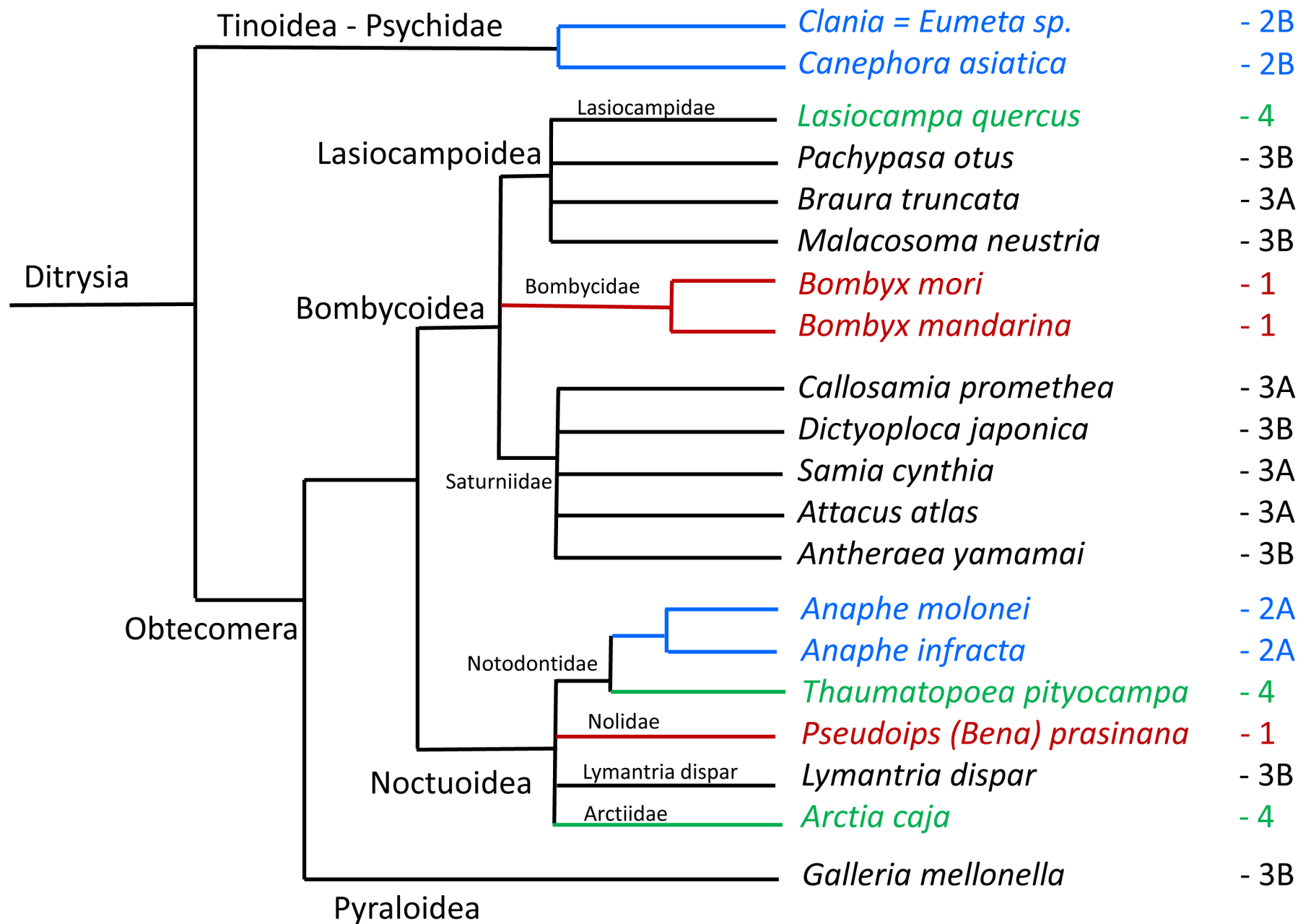

Supplement: Supplementary file 1 [file ijms-22-08246-s001.zip › Figure S1.pdf]
